# RISKS, REASONS AND RIGHTS: THE EUROPEAN CONVENTION ON HUMAN RIGHTS AND ENGLISH ABORTION LAW

**DOI:** 10.1093/medlaw/fwv020

**Published:** 2015-11-06

**Authors:** Rosamund Scott

**Affiliations:** Centre of Medical Law and Ethics, The Dickson Poon School of Law, King's College London, London, UK

**Keywords:** Abortion Act 1967 (as amended by the Human Fertilisation and Embryology Act 1990), European Convention on Human Rights, Article 8, Reform, Rights

## Abstract

Although there is no right to abort in English law but rather abortion is a crime, the lawful grounds for which are instantiated in the Abortion Act 1967 (as amended by the Human Fertilisation and Embryology Act 1990), the regulation of abortion is sometimes perceived as being fairly ‘liberal’. Accordingly, the idea that aspects of English law could be criticised under the European Convention on Human Rights, with which the UK must comply following the Human Rights Act 1998, may seem unlikely. Indeed, English law is compatible with the consensus amongst contracting states that abortion should be available on maternal health grounds. However, analysis of the UK's negative obligations under Article 8 shows that section 1(1)(a) of the Act is problematic as it operates in the first trimester. Further, given the European Court of Human Rights' emphasis on the reduced margin of appreciation once a state has legalised abortion to some degree and its jurisprudence relating to a state's positive obligations, the analysis shows that, while English law may not be problematic in relation to the lack of guidelines relating to the lawful grounds for abortion, it may well be in relation to the lack of a formal system for the review of any two doctors' decision not to grant a termination. Notwithstanding the morally serious nature of the decision to abort, the analysis overall raises questions about the need for at least some degree of abortion law reform, particularly in relation to the first trimester, towards a more autonomy-focused, though time-limited, rights-based approach.

## I. INTRODUCTION

Contrary perhaps to public perception, there is no right to abort in English law, at least if a right is understood as implying the freedom to exercise a choice for any or no reason.^1^ Rather, abortion is a crime, the lawful grounds for which are instantiated in the Abortion Act 1967 (as amended by the Human Fertilisation and Embryology (HFE) Act 1990).^2^ Accordingly, abortion is lawful only on the basis of certain grounds, or reasons. Despite this, the regulation of abortion in England is sometimes perceived as being fairly ‘liberal’.^3^ Indeed, some relatively recent press and political attention has criticised it in this regard.^4^ For this reason, it may seem unlikely that aspects of English law could be the subject of criticism under the European Convention on Human Rights (ECHR), with which the UK must comply following the Human Rights (HR) Act 1998. Indeed, English law is compatible with the consensus amongst contracting states that abortion should be available, for instance, on maternal health grounds.^5^ However, analysis of the negative and positive obligations of the UK under Article 8—the right to respect for private and family life—shows that English law may yet be vulnerable to challenge.^6^ Notwithstanding the morally serious nature of the decision to terminate a pregnancy, this raises questions about the need for at least some degree of abortion law reform, for instance towards a time-limited rights-based approach.^7^

The article focuses on three key issues. First, it considers whether the section of the Act which is employed as the legal basis for most UK abortions, the majority of which are performed in the first trimester of pregnancy, is compatible—specifically as it applies to the first trimester—with the UK's negative obligations under Article 8.^8^ This section renders abortion lawful where, in ‘good faith’, two doctors judge ‘that the pregnancy has not exceeded twenty-four weeks and that the continuance of the pregnancy would involve risk, greater than if the pregnancy were terminated, of injury to the physical or mental health of the pregnant woman or any existing children of her family’. The analysis particularly addresses the so-called ‘statistical argument’—which holds that the grounds for termination under section 1(1)(a)^9^ will almost always be fulfilled in the first trimester, and questions whether the requirements of section 1(1)(a), as the main condition of access to lawful abortion in the first trimester, constitute a justified interference with the right to respect for private life under Article 8. While the statistical argument's relevance to section 1(1)(a) has received a certain degree of academic, professional and political attention, all of which are considered here, the analysis is the first to address the argument in relation to the UK's human rights obligations.

Second, in the light of the criminal regulation of abortion and the relatively recent press, political and governmental attention to its regulation and provision,^10^ particularly under section 1(1)(a), the article draws on recent European Court of Human Rights (ECtHR) abortion case law to consider aspects of the UK's positive obligations under Article 8, addressing recent Crown Prosecution Service (CPS) findings and Department of Health (DH) statements and policy positions in this process.^11^ It asks, first, whether the UK has an obligation to produce guidelines for doctors as to when the lawful conditions for abortion may be met, again with particular reference to section 1(1)(a). It also asks whether the UK is compliant with Article 8, given the absence of any formal mechanism of review of any two doctors' decisions not to grant a termination. While the ECtHR's attention to these issues has tended to be cast in the literature, and to some extent by the Court itself, in a predominantly procedural light,^12^ the issues of guidelines and review may readily affect the substantive question of access to abortion.

Third, in the light of the analysis of the first two issues, the possibility of at least some degree of abortion law reform, particularly in relation to section 1(1)(a) and the first trimester, is raised. A move to a time-limited rights-based approach would respond to highly important recommendations in a Resolution of the Parliamentary Assembly of the Council of Europe from 2008, which have been described as ‘groundbreaking’.^13^

Before turning to consider the relationship between the ECHR and aspects of English abortion law, the article first gives a brief background section on the Court's approach to Article 8 in the abortion context.

## II. THE ECHR, ARTICLE 8 AND ABORTION

The ECHR jurisprudence on abortion has its origins in decisions of the European Commission, which addressed the issue of Article 8, in relation to the pregnant woman, and Article 2 (the right to life) in relation to the foetus.^14^

It is worth briefly noting the first case to consider the issue of abortion under the ECHR, *Brüggemann and Scheuten v Germany*,^15^ because it is still cited as an important case by the Court.^16^ In *Brüggemann* the Commission held (in part), that ‘pregnancy cannot be said to pertain uniquely to the sphere of private life. Whenever a woman is pregnant, her private life becomes closely connected with the developing foetus'.^17^ This passage is somewhat opaque. However, as the dissenting Judge Fawcett implied in his assessment of the majority judgment, the Commission appears to have held that since pregnancy is not only part of private life, regulation of pregnancy does not constitute an inference into private life and thus does not have to be justified under Article 8(2).^18^

Despite being a decision of the Commission, *Brüggemann* has played a prominent role in the ECtHR's abortion jurisprudence, typically being mentioned in a respectful light by the Court as forming the backdrop to its own analyses of abortion law. At the same time, the lack of clarity in the Commission's reasoning appears over time to have enabled the Court subtly but significantly to shift its position on the applicability of Article 8 to pregnancy. Importantly, despite recalling *Brüggemann*, the Court's more recent analyses appear now to have evolved into a position in which the regulation of abortion *does* constitute an interference with private life and, accordingly, must be justified under Article 8(2). For instance, in the important recent decision of *A, B & C v Ireland* the Court observed^19^:

The Court has previously noted, citing with approval the case-law of the former Commission in *Brüggemann* … that not every regulation of the termination of pregnancy constitutes an interference with the right to respect for the private life of the mother *(Vo v France* …). *Nevertheless*, having regard to the broad concept of private life within the meaning of Article 8 including the right to personal autonomy and to physical and psychological integrity … , the Court finds that the prohibition of the termination of the first and second applicants' pregnancies sought for reasons of health and/or well being *amounted to an interference with their right to respect for their private lives*.

In this passage, the Court appears relatively openly to shift the analysis from that of *Brüggemann*, as evidenced by its use of ‘[n]evertheless' and clearly states that abortion regulation must be justified under Article 8(2). Adopting a somewhat different method, in *RR v Poland*, decided the year after *A, B & C*, the Court appears instead to *reinterpret Brüggemann*, observing^20^:

The Court has also held that the notion of private live [*sic*] applies to decisions both to have or not to have a child or to become parents (*Evans v the United Kingdom* …). The Court has previously found, citing with approval the case-law of the former Commission, that the *decision of a pregnant woman to continue her pregnancy or not belongs to the sphere of private life and autonomy*. Consequently, also legislation regulating the interruption of pregnancy touches upon the sphere of private life, since whenever a woman is pregnant her private life becomes closely connected with the developing foetus.

Here *Brüggemann* appears to be recast as a decision that would have implied that the regulation of pregnancy has to be justified under Article 8(2), the position in fact held by the dissenting Judge Fawcett in that case. In short, as its other relatively recent jurisprudence—for instance in *Tysiąc v Poland*—also shows,^21^ it is now clear that the Court holds that abortion regulation amounts to an interference in the right to respect for private life under Article 8(1) and must therefore be justified under Article 8(2).

As for the nature of the interests in private life that the Court has recognised in this context, as can be seen above, in *A, B & C* the Court noted that it has ‘regard to the broad concept of private life within the meaning of Article 8 including the right to personal autonomy and to physical and psychological integrity’; and in *RR v Poland* it observes that ‘the decision of a pregnant woman to continue her pregnancy or not belongs to the sphere of private life and autonomy’. While the relationship between privacy and autonomy has long been the subject of debate in the literature,^22^ the Court is prepared to recognise a range of interests, including those in autonomy, under Article 8. Although it has acknowledged autonomy interests in the abortion context in its jurisprudence, such interests have not so far received as prominent an emphasis as they have in other medical law contexts, such as that of its jurisprudence in relation to assisted suicide.^23^ Further, in some abortion cases the interest in physical integrity has played a particularly prominent role, such as in *Tysiąc v Poland*, perhaps particularly given the issues at stake in that case, which related to termination on physical maternal health grounds, discussed below.

Focusing on section 1(1)(a) of the Act as it operates in the first trimester, the analysis turns shortly to consider whether English abortion law is Convention-compliant. Prior to this, some brief background relating to negative and positive obligations in turn, with particular reference to recent ECtHR abortion jurisprudence, is given.

### A. Negative and Positive Obligations: Blurred Distinctions and the Narrowing of the Margin of Appreciation

Convention states are under a negative obligation not unjustifiably to interfere with the right to respect for private and family life and a positive obligation to respect that right. The Court purports to use the terms ‘interference’ and ‘respect’ in quite distinct ways that map onto a state's negative and positive obligations in turn. In the first case, the question is essentially whether a given arrangement amounts to an unjustified (negative) interference with, say, the right to respect for private and family life. In the second, the question is whether a given set of legal arrangements respects someone's ability to exercise that legally recognised right or to realise that legally recognised interest. Thus, as the discussion will show, a (positive) failure of respect for an interest or right in fact may affect a person's substantive ability to exercise a right or to realise an interest, just as does a (negative) interference with a right. It may not be surprising then, that the Court has noted that the boundaries between negative and positive obligations ‘do not lend themselves to precise definition’.^24^

Turning first to the Court's approach to the analysis of a state's *negative* obligations under Article 8, as the Court frequently reiterates, whether an interference is justifiable under Article 8(2) involves a three-step analysis which considers whether the measure is in accordance with law, has a legitimate aim, and is necessary.^25^ The third element in turn hinges on a three-step assessment, established in *Sunday Times v United Kingdom*,^26^ that concerns whether there is a pressing social need for the interference, whether it is proportionate to the legitimate aim (there are conceptual links here with the idea of a pressing social need) and whether there are relevant and sufficient reasons for it. A detailed explication of these points can be found elsewhere.^27^ The notion of proportionality between the interests and rights of the individual on the one hand and those of the community on the other, including the public interest, is fundamental in this assessment and underlies the Convention as a whole. Thus, in *Soering v United Kingdom*, the ECtHR observed: ‘Inherent in the whole of the Convention is a search for the fair balance between the demands of the general interest of the community and the requirements of the protection of the individual's human rights’.^28^

As for the Court's analysis of a state's *positive* obligations under Article 8, in the abortion case of *Tysiąc v Poland* the ECtHR reiterates some general background of relevance here. It observes of the state's positive obligations^29^:

[T]hese … may involve the adoption of measures designed to secure respect for private life even in the sphere of relations between individuals, including both the provision of a regulatory framework of adjudicatory and enforcement machinery protecting individuals' rights and the implementation, where appropriate, of specific measures.

As in the negative context, the boundary with which the Court acknowledges is not precise, the Court must attend to the ‘fair balance’ between the interests of the individual and those of the community and, once again, the state has a certain margin of appreciation.^30^ As the Court has acknowledged, the notion of ‘respect’ is not clear, particularly regarding a state's positive obligations and, ‘having regard to the diversity of the practices followed and the situations obtaining in the Contracting States, the notion's requirements will vary considerably from case to case’.^31^ Nevertheless, the Court emphasises that the requirements of the rule of law, ‘inherent’ in all the Convention Articles, must provide a degree of protection against arbitrary interference with Convention rights. The idea that rights should be ‘practical and effective’, rather than ‘theoretical or illusory’ is also emphasised in *Tysiąc* and is of relevance likewise in the UK context.^32^ In *A, B & C v Ireland*, the Court notes that ‘certain factors' have been thought relevant in determining the content of a state's positive obligations and that as regards the applicant, there is the question of how important the interest at stake is, and whether ‘“fundamental values” or “essential aspects” of private life are in issue’.^33^

In *Tysiąc* itself, the Court decided to assess the applicant's claim that she had been denied access to an abortion with particular reference to the state's positive obligations to respect her interest in physical integrity.^34^ The issues in this case concerned the lack of a review procedure regarding the denial of the applicant's request for an abortion on health grounds, coupled with the lack of guidelines to determine whether the conditions for lawful abortion were met. These—and their implications for the UK—are discussed below. For present purposes, what emerges strongly from *Tysiąc* is that, even if states may be permitted a certain margin of appreciation in relation to the regulation of abortion, once a state has established a particular legal regime, the margin of appreciation *narrows* because the state can be held to account for not fulfilling its obligations within that regime.^35^ This is a very important point, which relates to the Court's wish to ensure, as noted above, that rights are not just hypothetical, but can be exercised in practice.^36^ As described by Fenwick, for instance, with reference both to *Tysiąc* and to *RR v Poland*, ‘the Court took the stance that where the state has provided for a degree of access to abortion to be available in law, the state's margin of appreciation does *not* extend to the *manner* in which it is made available’.^37^ The net result is that a state's legal arrangements may then be subject to what Fenwick has referred to as ‘more intensive scrutiny’, with reference also to *P & S v Poland*.^38^

The discussion now turns to consider the implications of these points for the UK, focusing on the interpretation and operation of section 1(1)(a) of the Abortion Act.

## III. ENGLISH ABORTION LAW: ARTICLE 8 AND SECTION 1(1)(A) OF THE ABORTION ACT

The termination of pregnancy in England is a crime under the Offences against the Person Act 1861.^39^ However, abortion is legal under section 1 of the amended Abortion Act 1967 if two doctors judge in ‘good faith’^40^:

(a) that the pregnancy has not exceeded its twenty-fourth week and that the continuance of the pregnancy would involve risk, greater than if the pregnancy were terminated, of injury to the physical or mental health of the pregnant woman or any existing children of her family; or (b) that the termination is necessary to prevent grave permanent injury to the physical or mental health of the pregnant woman; or (c) that the continuance of the pregnancy would involve risk to the life of the pregnant woman, greater than if the pregnancy were terminated; or (d) that there is a substantial risk that if the child were born it would suffer from such physical or mental abnormalities as to be seriously handicapped.

My focus here is particularly on the first of these—section 1(1)(a)—and especially as it applies in the first trimester of pregnancy because, as noted earlier, most terminations are performed under this ground before 12 weeks.^41^ What is the nature and purpose of the criteria in section 1(1)(a)?

These criteria have been described by the British Medical Association (BMA) as ‘medical’ justifications which, at one level, they are.^42^ By implication, this description would also apply to the grounds in sections 1(1)(b) and (c). Legally, the criteria in section 1(1)(a) render lawful conduct that would otherwise be a crime for the first 24 weeks of pregnancy. Morally, they reflect the view that such risks to a pregnant woman justify the termination of foetal life. The analysis now turns to consider section 1(1)(a) in the light of the UK's negative and positive obligations under Article 8, respectively.

### A. Negative Obligations: the Question of the Justifiability of the Interference

As far as the UK's negative obligations in relation to the ECHR are concerned, the key question in relation to the operation of section 1(1)(a) in the first trimester is whether the criminal prohibition on the termination of foetal life unless there is a risk to the woman's mental or physical health in going to term that is greater than in termination constitutes a justifiable interference with her right to respect for her private life.

In the abortion context, for instance in *Tysiąc*, the ECtHR has recently focused on the right to respect for private life as being instantiated in the interest in physical integrity. Although, as noted above, autonomy interests do receive brief mention in the cases,^43^ the Court appears to date to have underplayed somewhat the importance of autonomy in the abortion context, which it has recognised in other contexts, emphasising instead the question of health interests, a point also noted by others, such as Fenwick and Zampas and Gher.^44^ Accordingly, currently at least, the UK's negative obligations in relation to the right to respect for private life in the abortion context most clearly—but arguably not only—hinge on recognising a certain degree of a woman's interests in physical and psychological integrity, so that her right to respect for her private life is not unjustifiably interfered with. The question of ascertaining what the law recognises with regard to these negative obligations under section 1(1)(a) requires a certain degree of interpretation, to which I turn below. Whether the UK then respects/protects what *is* recognised is essentially a question about whether it is fulfilling its positive obligations, also discussed below.

#### 1. The Aim of Criminalisation and the Corresponding Lawful Ground in Section 1(1)(a)

Turning to the *first* question under the three-step analysis of the justifiability of an interference under Article 8(2), since abortion is regulated by two statutes (one establishing the crime, the other the lawful grounds), there is no question that the English provisions are in accordance with law. As for the *second* question—namely the legitimate aim of the establishment of the crime of abortion, the lawful grounds for which are those in section 1(1)(a) of the Act—this is not so clear. Legitimate aims of possible relevance under Article 8(2) include ‘the protection of morals' and the ‘protection of the rights and freedoms of others'.

In its report, *Scientific Developments Relating to the Abortion Act 1967*^45^ (which gave considerable attention to section 1(1)(a) and its operation in the first trimester) the House of Commons Science and Technology Committee recorded a number of reasons ‘that have been given for the introduction of the requirement for two doctor's signatures' in the Abortion Act, as follows^46^:

[T]o ensure that the provisions in the legislation were being observed; to protect women; to protect doctors from breaking the law; to demonstrate the medico-legal concerns of Parliament, namely that the 1967 Act did not make abortion legal but conferred upon doctors a defence against illegality—the two doctors are expected to police each other; to show the seriousness of the decision to terminate; and to appease the pro-life lobby.

It is unclear whether the Committee is concerned with the criminalisation of abortion *per se*, or the mechanism by which the lawful grounds to the crime are ‘policed’.^47^ However, since my concern goes beyond the ‘policing’ issue to that of the criminal nature of the regulation of English law, particularly as it plays out in the first trimester, it is the penultimate of these reasons, the notion of ‘show[ing] the seriousness of the decision to terminate’, that may be particularly relevant here. (An evaluation of the ‘politics' of the Act that may have been at stake in the last reason is beyond my scope.) The BMA has also noted that some have argued that the criteria may encourage women not to undertake abortion ‘lightly’.^48^ So, focusing on section 1(1)(a), one purpose of the criminal regulation of abortion could be the aim of encouraging some kind of ‘respect’ for foetal life, so that terminations are not undertaken, as it were, without due moral reflection. This could be thought to be relevant to the aim of the ‘protection of morals' under Article 8(2). A broader expression of some kind of societal respect for the foetus may well also be at stake.

Turning to the question of the pregnant woman's relationship with the foetus, people obviously disagree about whether, and if so how much, foetal life should be respected and also about what this would entail.^49^ However, as a matter of practice, although doctors may choose to discuss the criteria with women, there are no guidelines requiring them to do so when women present with their concerns and reasons.^50^ In any event, Joanna Erdman has noted that it is ‘widely reported that most women have reached a firm decision on whether to continue or terminate pregnancy before seeking health services'.^51^ Accordingly, it is unclear that this ground will necessarily serve the purpose of encouraging respect for foetal life on the woman's part, by means of serious consideration of it, notwithstanding that a woman will discuss her reasons with a doctor. This does not preclude the possibility that the woman would herself have given serious moral consideration to the question of termination prior to approaching a doctor, independently of the terms of legal access to abortion. Sally Sheldon has likewise noted this, suggesting that women may well ‘agonise’ over the decision.^52^

In the alternative, but perhaps more particularly in addition—since this would also be compatible with the notion of ‘respect’ for foetal life—the purpose of the crime and the corresponding lawful grounds under section 1(1)(a) could be the more straightforward one of reducing the number of terminations that might otherwise occur if the crime and the lawful grounds were not in place, that is, of protecting foetal life itself. In this respect, the foetus would have to be an ‘other’ under the aim of the ‘protection of the rights and freedom of others' under Article 8(2) (which does not require that the ‘other’ possesses Convention rights). Is the criminal structure of the law *necessary* to achieve this aim? This takes us to the *third* question entailed in the three-step analysis of the justifiability of the interference.

#### 2. Whether Criminalisation and the Corresponding Lawful Ground in Section 1(1)(a) are Necessary

This is a question about proportionality and entails assessing whether a ‘fair balance’ has been struck between the interests of the individual and those of the community, including the public interest.^53^ Thus, is there a sufficiently strong public interest in reducing the incidence of terminations to justify interference with a woman's right to private life and, in this context, particularly her interest in physical and psychological integrity? As noted earlier, under this third limb of the assessment of the justifiability of an interference, Convention jurisprudence requires that we consider, first, whether there is a ‘pressing social need’ for this legal arrangement. If we assume that the actual protection of foetal life is a legitimate aim, a key question may be whether foetal life would be more frequently terminated if abortion were not a crime with a corresponding lawful ground under section 1(1)(a). Thus, the number of abortions that would occur with and without this legal arrangement appears relevant to the question of a ‘pressing social need’. This raises the question of the extent to which doctors comply with requests and consider that the criteria are satisfied. In turn, this requires that we consider the degree of scope for interpreting the criteria and so the extent to which the criteria in fact limit access to abortion.

#### a. Interpreting the Criteria in Section 1(1)(a)

The term ‘mental and physical health’ is open to interpretation. The Royal College of Obstetricians and Gynaecologists (RCOG) refers in its guidance to the World Health Organisation's (WHO) definition of health as ‘… a state of physical, mental and social wellbeing and not merely an absence of disease or infirmity’ and observes^54^:

In determining whether there is a risk to mental health in a particular pregnancy the medical practitioners have to identify factors in the woman's life and personality that would threaten her mental health if the pregnancy were to continue: they do not have to certify that she has a mental illness.

The law on its face implies that it will be within doctors' discretion to evaluate a woman's *reasons* for requesting a termination and the *risks* to her of going to term versus termination, so that a termination may or may not be granted. Understood and applied in this way, the crime and the section 1(1)(a) criteria have the ability to affect the *number* of abortions by filtering out cases that doctors may consider do not satisfy the criteria. Doctors may vary in their reactions to such requests, some taking a more liberal approach than others.

If doctors *were* to deny access to termination on a significant number of occasions, the incidence of termination would be significantly reduced, so that one might conclude that there was a pressing social need for this discretion. However, there is also the question as to whether the interference in the right to respect for private life is proportionate to the legitimate aim of reducing the incidence of abortion (recalling the conceptual links with the idea of a pressing social need), as well as that of whether there are relevant and sufficient reasons for it. The effect on the number of terminations, about which empirical evidence would be required, would appear to be relevant at each stage of the ‘necessity analysis', as may be questions of the weight to be accorded to foetal life. It might be noted, however, with reference to criminal abortion laws generally, that Erdman has observed that there is no correlation between abortion rates and the legal status of abortion, noting that criminalisation does not result in the prevention of abortion.^55^ Further, this conclusion has been endorsed by the Parliamentary Assembly of the Council of Europe.^56^

Accordingly, leaving this strand of analysis aside, the discussion now turns to address the argument that, statistically, the grounds for termination under section 1(1)(a) (and section 1(1)(c) in fact) will automatically be fulfilled in the first trimester. While the ‘statistical argument’ has received prior professional, academic and political attention, it has not been previously considered in the context of a human rights analysis. I turn first to consider the attention the argument has received to date.

In a briefing paper entitled *First Trimester Abortion*, the BMA Medical Ethics Committee (MEC) notes that ‘evidence shows that the risks involved in first trimester abortion, particularly medical abortions, are less than the risks associated with carrying a pregnancy to term’, adding that ‘[i]n practice, therefore, few, if any, women will fail to meet the medical criteria in the first trimester’.^57^ The MEC goes on to state that ‘there are always legal grounds for abortion … because the risk to the life, and physical and mental health of a pregnant woman, of continuing a pregnancy, although low, will always be greater than the risk of terminating a pregnancy …’, citing Ian Kennedy and Andrew Grubb.^58^ Writing separately on this point, Andrew Grubb has argued that this must be so, unless there are reasons in a given woman's case to ‘upset the “statistical argument”’, adding that ‘[u]ltimately, the doctors must form an opinion that the ground applies to this individual and not solely on the basis of abstract statistics'.^59^

In this light, the BMA briefing paper goes on to state that ‘[t]he MEC believes that the requirement for medical criteria should be removed from first trimester abortions', and that it ‘believes that the requirement for two doctors' opinions should be removed for abortions within the first trimester’.^60^ In its evidence to the House of Commons Science and Technology Committee, the BMA stated^61^:

The BMA believes that the Abortion Act 1967 should be amended so that first trimester abortion (abortions up to 13 weeks) is available on the same basis of informed consent as other treatment, and therefore without the need for two doctors' signatures, and without the need to meet specified medical criteria. From a clinical perspective abortion is better carried out early in pregnancy. Given the relative risks of early abortion compared with pregnancy and childbirth, *virtually all women seeking an abortion in the first trimester will meet the current criteria for abortion*. The proposed amendment would help ensure that women seeking abortion are not exposed to delays, and consequently to later, more costly and higher risk procedures.

It also provided further details of the relevant evidence.^62^ The RCOG likewise argued in favour of this point in response to the Committee's enquiry, observing that^63^:

[W]omen in the first trimester could be seen as *automatically fulfilling the criteria* of [section 1(1)(a) of] the Abortion Act. Although this was not the original intention of the Act, in practice it facilitates access to induced abortion within the current law.

More recently, the British Pregnancy and Advisory Service (BPAS) has also supported this interpretation.^64^ We might call this the ‘automatic interpretation’.

Significantly, this argument was accepted by the House of Commons Science and Technology Committee, which included a review of the above-noted arguments of the BMA and the RCOG. With reference to the automatic interpretation, the Committee stated that ‘[t]here were dissenters to this view, but we found strong evidence that ground C is *always met* for first trimester abortions'.^65^ It then concluded^66^:

We were not presented with any good evidence that, at least in the first trimester, the requirement for two doctors' signatures serves to safeguard women or doctors in any meaningful way, or serves any other useful purpose. We are concerned that the requirement … may be causing delays in access to abortion services. If a goal of public policy is to encourage early as opposed to later abortion, we believe there is a strong case for removing the requirement for two doctors' signatures. We would like [*sic*] see this requirement … removed.

In short, while purporting to give doctors discretion in the interpretation and application of the criteria under section 1(1)(a), the criteria could be interpreted as having pre-determined the legal balance between a pregnant woman and the foetus in the first trimester. It is unlikely that Parliament was aware that the criteria might be interpreted in this way in relation to the first trimester since this would obviate the need for the crime, the criteria in section 1(1)(a) and the two doctors' signatures.

There are highly important and previously unconsidered implications of this interpretation for the question of the justifiability of the interference posed by section 1(1)(a) of the Act—as it applies in the first trimester—into a woman's private life under Article 8. First, given that (as the BMA put it) ‘few, if any, women will fail to meet the medical criteria’, the crime and the corresponding lawful grounds in section 1(1)(a) cannot be said to respond to a pressing social need. Second, in the light of the medical evidence, requiring *all* women seeking abortion in the first trimester to be subject to the criteria is disproportionate to the legitimate aim of protecting foetal life (recalling the centrality of the notion of proportionality to the necessity test and the conceptual links with the notion of a pressing social need).^67^ Third, while the reasons for the crime and the lawful grounds in section 1(1)(a) as they apply in the first trimester are relevant to the aim of protecting foetal life, they do not appear sufficient to justify the interference with the right to respect for private life. Thus, since there will always (or almost always) be greater risk to the woman in going to term than in termination in the first trimester, to impose a requirement to this effect as a condition of access to abortion in all cases, is *unnecessary*. Accordingly, on the automatic interpretation (and leaving the other grounds of the Act aside), making abortion lawful in the first trimester on the basis of the grounds of section 1(1)(a) would not pass the justification test under Article 8(2). This is of considerable significance when most abortions are carried out under this ground of the Act before 12 weeks.

Arguably, this is a justifiable interpretation of the law. However, there are no official guidelines as to how section 1(1)(a) should be interpreted. Does the UK have a positive obligation to issue these?

### B. Positive Obligations: Respect, Guidelines and Review

The question in relation to the UK's positive obligations is whether the current legal arrangement sufficiently protects/respects a woman's interest in psychological and physical integrity, as recognised in section 1(1)(a). Against the criminal backdrop of English law, here the analysis addresses again, from a different light, the issue of the scope for the interpretation of section 1(1)(a) of the Act, with particular regard to the first trimester, followed by the question of the absence of any system of formal review of any two doctors' decision not to grant a termination.

Doctors performing abortions in England are at once performing a crime and judging (or relying on one (or two) other doctors' judgment) in ‘good faith’, that one or more of the lawful grounds under the Abortion Act is made out. The ECtHR commented in both *Tysiąc* and *A, B & C* on the effect that criminal sanctions may have on the provision of abortion, referring to the notion of a ‘chilling effect’ or ‘chilling factor’.^68^ The Court referred to two particular issues in both these cases: first, to the breadth—and potential uncertainty—of a provision establishing the legality of abortion; and second, to the question of the lack of a mechanism to review doctors' decisions not to offer terminations.^69^ These issues were prominent also in *RR v Poland* and *P & S v Poland*, as also noted below.

#### 1. Guidelines

Turning first to *A, B &C*, the Court noted the ‘broad’ terms in which the ground for legal abortion in Ireland is expressed, namely where ‘it is established as a matter of probability that there is a real and substantial risk to the life, as distinct from the health, of the mother, including a risk of self harm [*sic*], which can only be avoided by a termination of the pregnancy’.^70^ It observed that although ‘a constitutional provision of this scope is not unusual, no criteria or procedures have been subsequently laid down in Irish law, whether in legislation, case law or otherwise, by which that risk is to be measured or determined, leading to uncertainty as to its precise application’.^71^ The Court further noted that ‘the guidelines do not in any event provide any relevant precision as to the criteria by which a doctor is to assess the risk’.^72^ In the light of this ‘background of substantial uncertainty’ the *A, B & C* Court held that the criminal structure of Irish law ‘would constitute a significant *chilling factor* for both women and doctors in the medical consultation process',^73^ independently of whether there had in fact been any prosecutions. In *Tysiąc*, the Court likewise drew attention to ‘a chilling effect on doctors when deciding whether the requirements of legal abortion are met in an individual case’.^74^ The Court observed: ‘The provisions regulating the availability of lawful abortion should be formulated in such a way as to alleviate this effect’; and it noted that ‘[o]nce the legislature decides to allow abortion, it must not structure its legal framework in a way which would limit real possibilities to obtain it’.^75^ Importantly, here it emphasises the significant *reduction in the margin of appreciation* once a state has permitted abortion under certain terms.

In England, too, there has been a dearth of prosecutions. The notable exception as regards doctors is *R v Smith*, in which it was found that the doctor had not formed a good-faith opinion with regard to section 1(1)(a) of the Act and had not sought a second opinion before agreeing to terminate, despite alleging at trial that this was provided by the anaesthetist who in fact only saw the woman just before the procedure.^76^ The idea that uncertainty about the interpretation of the criteria could result in a ‘chilling effect’ not just in Ireland, but also in England, may seem unlikely given that English abortion law is much more liberal in that it permits termination on maternal health grounds. However, while awareness of the fundamentally criminal nature of abortion may be less widespread among the public than in Ireland, doctors are inevitably aware of this facet of the law and could, therefore, be subject to a ‘chilling effect’. The extent to which this could occur will likely turn on the climate in which doctors are currently operating, notably whether this is one in which abortion is publicly perceived as a procedure that should be relatively easily available, at least in the first trimester, or whether the accessibility of abortion is perceived as a matter of public concern, which could in turn affect at least some doctors in their interpretation of the criteria in section 1(1)(a).

Of significance in relation to this point, relatively recently there was press coverage about the possibility of terminations being authorised on the grounds of foetal sex which, by *itself*, is not a ground for termination under English law, but which might well qualify under section 1(1)(a).^77^ Certain clinics were investigated, also in relation to signing practices of the relevant ‘HSA/1’ and ‘HSA/2’ forms.^78^ Subsequently, in February 2012 the Chief Medical Officer (CMO) sent a letter to doctors reiterating the legal grounds, as they appear in the Act, for termination.^79^ Meanwhile, the General Medical Council (GMC) put a statement on its website that was subsequently criticised (by letter to the GMC) by a number of medical law professors for being ‘erroneous and misleading in important respects'; it was suggested in that letter that the GMC should refer doctors to the CMO's letter and the GMC subsequently did this.^80^ In the light of this renewed attention to section 1(1)(a) of the Act, BPAS stated in May 2013 that ‘doctors involved in abortion care have become nervous about their everyday practice, as it no longer seems clear what is legal and illegal, or which aspects of standard abortion practice may be suddenly highlighted as problematic by the regulators'.^81^ Thus, despite the apparently liberal face of English abortion law, these developments highlight the potential for the criminal nature of abortion regulation to bear heavily on practice.

In October 2013 the CPS subsequently decided not to prosecute two doctors following investigations into apparent requests for terminations on the grounds of foetal sex, on the basis that there was insufficient evidence to prosecute to the criminal standard and that it was not in the public interest.^82^ The CPS noted (in part) that^83^:

[T]here was no guidance on how a doctor should go about assessing the risk of [*sic*] physical or mental health, no guidance on where the threshold of risk lies and no guidance on a proper process for recording the assessment carried out. The *discretion* afforded to a doctor in assessing the risk to the mental or physical health of a patient wanting an abortion is *wide* and, having consulted an experienced consultant in Obstetrics and Gynaecology, it appears that there is no generally accepted approach among the medical profession.

Referring to the CPS's decision, the CMO subsequently stated that the CPS ‘highlighted the lack of guidance around how both doctors should go about assessing the risk to the physical or mental health of the pregnant woman’, and that the government ‘will address these issues in revised guidance, while acknowledging the discretion allowed to doctors under the Act in reaching a decision in good faith and the role of clinical judgement’.^84^

Importantly, it is perhaps a matter of debate as to whether and, if so, to what extent ‘revised guidance’ is in fact required. While both *Tysiąc* and *A, B & C* highlighted the possible need for guidelines under a state's positive obligations, these decisions are by no means prescriptive as to the degree of uncertainty that would generate the need for guidelines or as to the requisite detail of such guidelines. The key *legal* point, as regards the UK's positive obligations, is that once a state has said that abortion is lawful on certain grounds, it must protect or guarantee the availability of abortion on those grounds, since its margin of appreciation in relation to abortion will have been significantly reduced.

As to what the lawful grounds for abortion are, on one view it might be thought that the CMO's letter of 23 February 2012, which was regarded positively by the medical law professors who wrote to the GMC, may be sufficient to explain the law,^85^ especially because the Abortion Act puts such weight on the good-faith opinion of doctors, a point necessarily noted by the CMO in that letter. This may be particularly so provided doctors are reassured as to the legal significance of their good-faith opinions, so that any possible ‘chilling effect’ is dispelled or avoided.^86^ Of note, the CPS also highlights the width of medical discretion (in the passage quoted above) and the significance of doctor's good-faith opinions to the operation of the Act, as in the passage below^87^:

The prosecution would have to be in a position to prove, beyond reasonable doubt, that the assessments carried out by the doctors was [*sic*] carried out in *bad faith* or carried out in such a way that fell below a standard which any reasonable doctor would consider adequate. In the absence of any considered *medical* guidance it is extremely difficult for the prosecution to undertake this exercise. Equally, it would be very difficult for a jury to assess what may or may not be an ‘adequate’ assessment by the doctor.

Of note, the CPS here refers to the notion of ‘medical’ guidance as opposed, for instance, to governmental guidance. Coupled with the CMO's acknowledgement (noted above in her letter of 22 November 2013) of ‘the discretion allowed to doctors under the Act in reaching a decision in good faith and the role of clinical judgement’, in fact this raises a very important point about the potential relationship between any governmental guidance regarding the grounds for legal abortion and the Abortion Act itself. Thus, as the CMO's letter might be thought to indicate, any governmental guidance must be compatible with the Abortion Act's emphasis on the good-faith opinion of two doctors, since it is their good-faith opinion which renders an abortion lawful under the Act.^88^

In this regard, and importantly, English law is notably different from the Irish law that the ECtHR considered in *A, B & C v Ireland* which, as observed above, requires ‘*as a matter of probability* that there is a real and substantial risk to the life … of the mother’.^89^ It is also different from the Polish law that the Court addressed in *Tysiąc v Poland*, which ‘provided that legal abortion was possible only until the twelfth week of pregnancy where the pregnancy endangered the mother's life or health’,^90^ with accompanying regulations stating that the ‘[t]he *circumstances indicating* that pregnancy constitutes a threat to the woman's life or health shall be *attested by* a consultant specialising in the field of medicine relevant to the woman's condition’.^91^ There is no reference in either case to any good-faith medical opinion. In contrast, highlighting the significance of good faith to the operation of the English Abortion Act, in *R v Smith*, Scarman LJ cited with approval part of the Recorder's (Judge Sir Carl Aarvold) summing up to the jury, as follows^92^:

If two doctors genuinely form an opinion in each case that they deal with that the risk of continuance is more than the risk of termination, *it does not matter whether they are right or wrong* in that view. If they form that opinion genuinely and in good faith, that in fact comes within the Act, and there is no guilt attached to it.

In May 2014, the DH issued its planned guidance, entitled *Guidance in Relation to Requirements of the Abortion Act 1967* (the *Guidance*).^93^ Interestingly, by way of ‘background’, this states^94^:

The guidance does not, and indeed cannot, change the law in relation to abortion, which is governed by the criminal law and the Abortion Act and is ultimately a matter for Parliament and the courts to determine. *However, the intention is to provide support for doctors by setting out how the law is interpreted by the Department of Health*.

On the question of ‘[f]orming an opinion in good faith’, the document states^95^:

If there is evidence that either certifying doctor has not formed their opinion in good faith then the doctor performing the termination is not protected by section 1(1) of the Abortion Act and has potentially committed a criminal offence by terminating the pregnancy. It is also possible that the doctor could be acting contrary to their professional duties.

The *Guidance* also notes that while there is ‘no legal requirement’ for at least one of the doctors to have seen the woman, the DH view is that this is ‘good practice’.^96^ On the question of certification, the *Guidance* observes that this ‘takes place in the light of … [the doctors'] clinical opinion of the circumstances of the pregnant woman's individual case’.^97^ As regards the ‘lawful grounds for abortion’, it notes that these are ‘set out’ in an Annex.^98^

The Act's and *Guidance*'s emphasis on doctors' good-faith opinion is relevant to the government's (and indeed Parliament's) need to consider the automatic interpretation argument (discussed above) that, as regards first-trimester terminations, the grounds for termination under section 1(1)(a) (and also, in fact, under section 1(1)(c)) of the Act will always, or almost always, be made out. Indeed, given that medical evidence and professional organisations such as the BMA and RCOG support this interpretation, it is not clear that it would be open to the government to reject it, given the Act's emphasis on good-faith *medical* opinion. The DH *Guidance* does not directly address this argument. Rather, with regard to the question of ‘[a]ssessing risk to physical or mental health, the threshold of risk and recording how the assessment is carried out’ (issues identified by the CPS, as noted above), it states^99^:

12. Whilst there is no statutory requirement for either doctor to have seen and/or examined the woman, it is the Department's interpretation of the law that both doctors should ensure that they have *considered sufficient information specific to the woman* seeking a termination to be able to assess whether the woman satisfies one of the lawful grounds under the Abortion Act.

13. This assessment will include consideration of any risk to the woman's physical or mental health as one of the lawful grounds. The identification of *where the threshold of risk to the physical or mental health of the woman lies is a matter for the clinical opinion* for each of the doctors.

14. Although the burden of proof would be on a prosecutor to show that an opinion was not formed in good faith, DH recommend that RMPs should be prepared to *justify how they considered information specific to the woman when forming their opinion*, for example by recording in the patient record that they have assessed the relevant information and reached the conclusion based on this information. This is in line with guidance from the GMC (see Annex B).

Importantly, this accepts that the ‘threshold of risk’ is a matter of clinical judgment. While the *Guidance* stresses the need to consider ‘sufficient information specific to the woman’, this is by no means inconsistent with the medical evidence (as detailed, for instance, by the BMA)^100^ and, in the light of that, the BMA's and RCOG's view that the ground will always be fulfilled, except if the doctors were to judge that there is something in a particular woman's circumstances to upset the statistical balance. (Recall here Andrew Grubb's argument and his point (cited earlier) that ‘[u]ltimately, the doctors must form an opinion that the ground applies to … [the woman] and not solely on the basis of abstract statistics'.) In other words, in the light of the medical evidence regarding risks, a doctor may well simply *confirm* (in fact) that the ground applies, provided nothing in a woman's particular circumstances changes the statistical balance (which is very unlikely). Importantly, attending to the relevant statistical risks would be consistent with the evidence-based approach to medicine that has become prevalent since the Act was passed.

Accordingly, since the criteria in section 1(1)(a) will always (or almost always) be satisfied, the government needs to consider my earlier conclusion, with reference to the ‘necessity’ analysis under Article 8(2), that the criminalisation of abortion in the first trimester is not a justifiable interference with a woman's right to respect for her private life under Article 8. Although the DH *Guidance* states that ‘[t]he purpose of the requirement that two doctors certify the ground(s) for termination is to ensure that the law is being observed; this provides protection for the woman and for the doctors providing the termination’,^101^ this cannot be relevant to the question of the *justifiability* of the current legal arrangement. In the *Government Response to the Report from the House of Commons Science and Technology Committee on the Scientific Developments Relating to the Abortion Act 1967* in 2007, with reference to the Committee's recommendation that the two-signature requirement should be removed, the government stated^102^:

We note the Committee's recommendations. The requirement for two doctors' signatures was believed necessary when the Abortion Act 1967 was passed, to ensure that the provisions in the 1967 Act were being observed and to safeguard women. The decision to require two doctors' signatures was based on professional opinion at the time. We note that both the British Medical Association and the RCOG believe that there is no need for two doctors' signatures in the first trimester, and this will be a consideration for Members of Parliament if this issue should come before Parliament.

In the light of the argument in this paper, the government now *also* needs to consider the compatibility of the two-signature requirement, coupled with the current criminalisation of abortion in the first trimester and the operation of section 1(1)(a) in that trimester, with Article 8 of the ECHR. The question of possible law reform is addressed below.

#### 2. Review

The *Tysiąc* Court observed that the ‘concepts of lawfulness' and the rule of law require that ‘measures affecting human rights be, in certain cases, subject to some form of procedure before an independent body competent to review the reasons for the measures and the relevant evidence’.^103^ It also stated that, with regard to the issue of abortion, ‘such a procedure should guarantee to a pregnant woman at least the possibility to be heard in person and to have her views considered’ by a ‘competent body’,^104^ which should provide written reasons. Building on *Tysiąc*, similar observations were made by the Court in *RR v Poland.*^105^ The Court in *A, B & C*, which raised a number of concerns about the ‘effectiveness'^106^ of the ‘ordinary medical consultation process between a woman and her doctor … as a means of establishing … [the woman's] qualification for a lawful abortion’,^107^ likewise criticised the Irish state, specifically in relation to the third applicant in that case, for the absence of a^108^:

[f]ramework whereby any difference of opinion between the woman and her doctor or between different doctors consulted, or whereby an understandable hesitancy on the part of a woman or doctor, could be examined and resolved through a decision which would establish as a matter of law whether a particular case presented a qualifying risk to a woman's life such that a lawful abortion might be performed.

It might be noted here that Ireland has now acted to rectify its breach in relation to the third applicant in that case by enacting the *Protection of Life During Pregnancy Act* 2013.^109^ (In contrast, as noted earlier, given that the first and second applicants were able to travel to England to obtain a lawful abortion, the Court found no breach under a negative analysis, thus permitting what Erdman has here described as a ‘harm reduction’ approach to bolster the highly restrictive nature of Irish abortion law.^110^)

One reason for a doctor to decline to agree to provide an abortion is that of conscientious objection. In *RR v Poland*, the ECtHR held^111^:

States are obliged to organise the health services system in such a way as to ensure that an effective exercise of the freedom of conscience of health professionals in the professional context does not prevent patients from obtaining access to services to which they are entitled under the applicable legislation.

Further, in *P & S v Poland* the Court gave careful consideration to whether the relevant ‘procedural requirements' had, in reality, been given proper effect and found that they had not been.^112^ Thus, the Court is prepared to consider this issue very closely.

Turning now to consider the issues of conscientious objection and review in the English context, and reflecting on the operation of the law also beyond the first trimester, the DH does not collect data on how many terminations are declined. At present, however, a woman who has not been granted a termination can seek to have her termination approved by other doctors. The House of Commons Science and Technology Committee heard evidence relating to the possibility of doctors' right of conscientious objection (which it did not question) creating delay in access to abortion.^113^ The Committee noted that ‘in the guidelines commissioned and promoted by the Department of Health, it is recommended that practitioners who conscientiously object should refer the patient as soon as possible to another doctor who does not conscientiously object’.^114^ The Committee stated that^115^:

Professional guidance is not as clear as this and we urge the General Medical Council, while preserving the right of doctors to conscientiously object and not to refer directly to another doctor for an abortion unless it is an emergency, to make clear that conscientious objectors should alert patients to the fact that they do not consult on abortions and that if the issue arises during a consultation that they have a duty immediately to refer the patient to another doctor for the consultation.

Where a doctor has a conscientious objection to abortion, current GMC guidelines (2008) now state that s/he must ‘tell patients of their right to see another doctor with whom they can discuss their situation and ensure that they have sufficient information to exercise that right’;^116^ moreover, that ‘if the patient cannot readily make their own arrangements to see another doctor [doctors] must ensure that arrangements are made, without delay, for another doctor to take over their care’.^117^ If doctors follow these guidelines, a woman will know that she has the chance at least to seek a termination elsewhere.

However, where a termination is refused and reasons of conscience are not present or cited, the important question arises as to whether a woman will be aware that doctors might interpret the grounds under the Act differently, particularly as they apply beyond the first trimester, rather than that a termination may simply not be available under the law. A great deal will turn on the knowledge and social awareness of the pregnant woman herself at this juncture.^118^ In some ways, seeking a second opinion would be an informal self-generated form of review of the first opinion(s) given. Whether a woman obtains a termination will turn on the view of the second doctor whom she consults (or third and so on if she is persistent enough). Such a system is vulnerable because of possible differences of opinion between doctors. Moreover, the implications of both *Tysiąc* and *A, B & C* are that this current system may breach the UK's positive obligations under Article 8, notwithstanding that the operation of English abortion law hinges on doctors' good-faith opinions in a way that the Irish and Polish law do not.

A review procedure would have to allow for a woman's views to be heard, and any hearing and decision would have to take place in a sufficiently timely fashion, given the importance of the avoidance of delay, a point noted by the *Tysiąc* court.^119^ As has been emphasised above, much would still turn in any review on what can be a good-faith interpretation and application of the criteria in section 1(1)(a), either throughout the first and second trimesters, if the automatic interpretation point regarding the first trimester were not accepted by the government or the courts (which, it has been suggested, would not in fact be open to them), or if it were, then only between 12 and 24 weeks. From the point of view of a woman seeking a termination, while a review procedure may be burdensome, it would not be as burdensome as the situation of a failure to secure a termination and no recourse to review. However, the question of legal alternatives that would be less burdensome, notably the idea of a right to abort *per se* (that is, one that does not have to be justified with regard to particular risks or reasons), at least in the first trimester, is briefly considered below.

Before I turn to consider reform of English law, I touch on the important question of to what extent the just-discussed issues of guidelines and review can be understood only as ‘procedural’ ones as others, such as Zampas and Gher, have described them.^120^ Indeed, the *Tysiąc* Court might be said itself to invite this description by noting that ‘[w]hile Article 8 contains no explicit *procedural* requirements, it is important for the effective enjoyment of the rights guaranteed by this provision that the relevant decision-making process is fair and such as to afford due respect to the interests safeguarded by it’.^121^ Of note, this reference by the majority to ‘procedural requirements' might be thought, perhaps for political reasons, to downplay the significance of the finding of a breach in *Tysiąc*. Indeed, Judge Bonello—in his Separate Opinion—stressed that he was prepared to find a violation simply on the basis that^122^

[t]he Court was only called upon to decide whether, in cases of conflicting views (between a pregnant woman and doctors, or between the doctors themselves) as to whether the conditions to obtain a legal abortion were satisfied or not, effective mechanisms capable of determining the issue were in place.

#### 3. The Relationship Between Substance and Procedure, Interference and Respect

An important question that arises in relation to a state's positive obligations regarding, for instance, respect for a woman's interests in psychological and physical integrity under Article 8, is to what extent respect for these interests has *substantive*, rather than, say, only *procedural* implications. In this regard, the partly dissenting Judge de Gaetano in *RR v Poland* criticised the majority both in *Tysiąc* and in *RR* for not analysing the cases under Article 6.^123^ In effect, this criticism raises the question of the relationship between substantive and procedural interests in this context. It also raises the question of the relationship between a state's negative and positive obligations, and the meaning of each of these in turn. There are a number of statements in *Tysiąc* that make the Court's position on this issue somewhat unclear.

Opening its consideration of Article 8, the Court observes^124^:

[T]he applicant complained that the facts of the case had given rise to a breach of Article 8 of the Convention. Her right to due respect for her private life and her physical and moral integrity had been violated both *substantively*, by failing to provide her with a legal therapeutic abortion, *and* as regards the State's *positive obligations*, by the absence of a comprehensive legal framework to *guarantee* her rights.

As the Court reports the applicant's argument, the term ‘substantively’ is implicitly used in conjunction with the State's *negative* obligations and the term ‘guarantee of rights' is used in conjunction with the State's *positive* obligations. Later, the Court observes that ‘the applicant submitted that the refusal of an abortion had also amounted to an interference with her rights *guaranteed* by Article 8’, but that ‘the Court is of the view that the circumstances of the applicant's case and in particular the nature of her complaint are more appropriately examined from the standpoint of the respondent State's … positive obligations alone’, apparently now using the term ‘guarantee’ in conjunction with the concept of negative obligations.^125^ The boundaries between the Court's reporting of the applicant's argument and its own statements are not fully clear in the passages quoted, but the Court appears to use the notion of ‘guaranteeing rights' both in relation to its negative and positive analyses. In this light, we might ask here why a failure of positive obligations does not amount to an ‘interference’ with a right, thus more overtly raising substantive issues?

Indeed, it is apparent that flaws in a legal regime governing abortion, such as the absence of a review procedure, can affect the substantive question of whether a woman is able legally to obtain an abortion to which the law says she is entitled. In other words, what might be seen as the ‘purely procedural’ issue of the absence of a review mechanism in this context in fact affects the substantive question of access to abortion. For this reason, the partly dissenting Judge de Gaetano's criticism that both *Tysiąc* and *RR v Poland* should instead have been analysed under Article 6 is not sustainable. Indeed, the effect of that argument would be to neglect the positive obligations that a state has under Article 8 in addition, that is, to its negative ones. As regards the legal position in Poland itself, what the Court effectively says in these cases is that the state failed positively to respect the legally protected interest in abortion that a woman has under Polish law. It is worth noting here that, unlike Ireland, Polish law on its face is compatible with the consensus view (noted earlier) that abortion should be available on maternal health grounds because it recognises maternal health interests as a legal ground for abortion.

Thus, while the Court's focus in these cases was ostensibly on procedural issues, the issues clearly have a bearing on the substantive question of access to abortion, as Fenwick—who notes that ‘a purely “procedural” reading does not fully exhaust the implications' of the cases—likewise suggests.^126^ This may also account for the disquiet of the dissenting Judge Borrego in *Tysiąc*, who went so far as to say that the Court contradicted itself when it said that ‘it is not the Court's task in the present case to examine whether the Convention guarantees a right to have an abortion’,^127^ and that by means of its findings the Court was, in effect, permitting ‘abortion on demand’.^128^ The potentially substantive implications of supposedly purely procedural flaws give further weight to the need for at least some degree of English law reform, to which I now turn.

### C. Law Reform

Currently, English abortion law might be said to be characterised by a degree of pragmatic compromise. Margaret Brazier and Susanne Ost have insightfully argued that this is expressive of a political liberal balance, realised by means of the criminal law, between conflicting moral views as to the respective strength of the pregnant woman's and the foetus's interests or claims.^129^ Despite this, the analysis in this article has shown that, as regards Article 8 of the ECHR, aspects of the law are also problematic in significant ways.

There are two possible responses to the problem, particularly, of the lack of justification for the requirements in section 1(1)(a) of the Abortion Act as this operates in the first trimester of pregnancy. First, the grounds for termination could be revised in some way, so that the statistical argument—under which ‘few, if any’ (per the BMA) women would not satisfy the criteria—no longer applies in the first trimester. In the alternative, access to termination of pregnancy in the first trimester (and potentially beyond) could be further liberalised so that women have a right to abort *per se*, sometimes known as a right to abortion ‘on request’. Others, such as Sally Sheldon, have long argued for this in insightful and important ways.^130^ Very importantly, if English law were to be reformed to grant a time-limited right to abortion, this would be in line with the Parliamentary Assembly of the Council of Europe's recommendations, back in 2008, in favour of the decriminalisation of abortion ‘within reasonable gestational limits' and the granting of ‘freedom of choice’ with regard to abortion in all states where this has not already occurred.^131^

If a woman were to have a time-limited right to abort *per se*, she would have the *right* to abort without being subject to an assessment—or rather a confirmation, in the first trimester—of the *risks* in her case, or of her *reasons*. (Of course, the latter could well have a bearing on her risks, particularly as regards the question of psychological integrity.) As noted earlier, the BMA recommended in evidence to the House of Commons Science and Technology Committee that ‘the Abortion Act 1967 should be amended so that first trimester abortion (abortions up to 13 weeks) is available on the same basis of informed consent as other treatment, and therefore without the need for two doctors' signatures, and without the need to meet specified medical criteria’.^132^ The BMA stressed that ‘[f]rom a clinical perspective abortion is better carried out early in pregnancy’ and, as noted earlier, drew attention to the statistical argument.^133^ It also stressed that ‘[t]he proposed amendment would help ensure that women seeking abortion are not exposed to delays, and consequently to later, more costly and higher risk procedures'.^134^

We have also seen that the House of Commons Science and Technology Committee found that it had not heard ‘any good evidence that, at least in the first trimester, the requirement for two doctors' signatures serves to safeguard women or doctors in any meaningful way, or serves any other useful purpose’.^135^ As noted, the Committee also observed that it was ‘concerned that the requirement … may be causing delays in access to abortion services'.^136^ Further, the Committee was not satisfied with the government's submission ‘that the high percentage (89%) of abortions that take place in the first trimester is an indicator that “there is not a problem”’.^137^ The DH has recently observed that ‘Department of Health policy is that women who are legally entitled to an abortion should have access to the procedure as soon as possible. Evidence shows that the risk of complications increases the later the gestation’.^138^ Thus, not only are the requirements of section 1(1)(a) as a condition of lawful access to most first-trimester abortions unjustifiable under Article 8(2) of the ECHR. In addition, in the event that the requirements were to delay access to termination beyond the first trimester, a woman would then undergo a more risky surgical termination. In the light of the argument in this paper, and very significantly, there would be no legal justification for the imposition of these increased risks. Thus, issues of harm reduction would also be importantly implicated in decriminalisation, for instance within a certain gestational limit.^139^

Further, as the House of Commons Science and Technology Committee stressed, reform would aid the ‘goal of public policy … of encourag[ing] early as opposed to later abortion’.^140^ Apart from the question of reduced maternal risks with early abortion, on the gradualist account of the foetus's legal status that is embedded in English abortion law (given that section 1(1)(a) no longer applies after 24 weeks),^141^ the law implicitly represents that the foetus's moral status increases during its gestation, thus potentially requiring greater justification for termination as pregnancy progresses. Thus, reform that helped to ensure that desired terminations occurred earlier rather than later would be in line with this position.

Rights-based approaches may have the down-side, in closely contested moral areas, of creating a polarising effect on public debate, as Brazier and Ost also note.^142^ Indeed, this itself might be thought to be a reason in favour of the compromise currently represented by English abortion law. For instance, the issue of abortion is heavily polarised in the rights-based context of the United States. However, this may have been so long before the Supreme Court established the right to abort in *Roe v Wade.*^143^ It may be particularly helpful to look elsewhere in Europe, where there are several countries, including Denmark and Sweden, that permit abortion ‘upon request’ until a certain gestational limit.^144^ If a right to abort were established in English law, the question would arise as to the duration of that right, and as to the implications for the interpretation of the law beyond the time-limit for its exercise. On one model, there could be a right until the end of the first trimester (13 weeks, per the BMA).^145^ The second trimester could then be governed by an arrangement similar to, or the same as, that which is now in place. Alternatively, a right could be granted until approximately 18 weeks (as in Sweden) or until the point of foetal viability—which has a moral significance that is also compatible with the notion of the foetus acquiring interests with the development of sentience (which is at about the same time)^146^—leaving the decision to a duly informed (as to medical risks and so forth) and, it would be hoped, morally reflective pregnant woman. The remaining grounds would continue to be relevant after 24 weeks.^147^

Overall, it would be very important that any reform, say, of first- or first- and second-trimester abortion law does not make access to abortion thereafter harder than it currently is, as for instance the BMA has also noted.^148^ The question of the political appetite for abortion law reform is, of course, another matter.^149^

## IV. CONCLUSIONS­

This article has considered the compatibility of aspects of English abortion law with the ECHR. The UK is a party to the European consensus under Article 8 that abortion should be available on maternal health grounds and not just, for instance, where there is a risk to the woman's life (as in Ireland). Yet it is not clear that English law is fully compatible with the Convention. While this may seem surprising, it is in accordance with the approach of the Parliamentary Assembly of the Council of Europe, which noted that while ‘[a]bortion is permitted in the majority of European countries for a number of reasons … [t]he Assembly is nonetheless concerned that, in many of these states, numerous conditions are imposed and restrict the effective access to safe, affordable, acceptable and appropriate abortion services', citing ‘repeated medical consultations' as an example.^150^

The relationship between the statistical argument and section 1(1)(a) of the Act has here been analysed for the first time in relation to human rights obligations. The analysis has shown that criminalising abortion in the first trimester unless there is a risk to the pregnant woman's physical or mental health of going to term that is greater than in termination is not justified under a negative analysis of the UK's obligation to respect private life, under the ‘automatic’ interpretation of that section. In short, since there is ‘strong evidence that [section 1(1)(a)] is always met for first trimester abortions' (per the House of Commons Science and Technology Committee, as noted above), to impose a requirement to this effect as a condition of lawful access to abortion in all cases (in relation to which the other grounds are not appropriate, that is) is *unnecessary*. Accordingly, seeking to make access to lawful abortion conditional on fulfilment of the terms of section 1(1)(a) would not pass the test to justify interference in a woman's private life under Article 8(2). Given that the majority of abortions are carried out under this ground of the Act before 12 weeks, this is highly significant. Moreover, as it stands, the operation of the law may result in some cases in delayed access to termination, coupled with the increased maternal risks of second-trimester surgical abortion, for which there would be no legal justification on the UK's part.^151^

Given the importance of good-faith medical opinion to the operation of the Act, it is doubtful if the UK is in breach of its positive obligations under Article 8 by failing to provide more detailed guidelines regarding the interpretation of the grounds in the Abortion Act. However, the lack of a system of formal review of any two doctors' decisions not to grant a termination is problematic and, while at one level a procedural issue, clearly has the potential to impact on the substantive question of access to abortion. Thus, regardless of the relatively liberal face of English abortion law, in practice access to termination could be illegitimately hampered—a position that cannot be protected by the margin of appreciation once a given legal arrangement is in place—thereby potentially putting the UK in breach of Article 8 and in tension with the Convention's intent that rights be real, and not illusory.

If a limited right to abort were established in English law this could be restricted, not by the question of the degree to which the interests in physical and psychological integrity must be invoked, with reference to maternal *risks* or *reasons* (as is necessary for instance in relation to the comparative exercise at stake in section 1(1)(a)), but rather by time-limits. In essence, this would of course be to prioritise an *autonomy*-focused interpretation of Article 8 in the abortion context over one which stresses the maternal interests in physical and psychological integrity, that is, the maternal *health* interests on which the majority of the lawful grounds for abortion are currently founded.^152^ At the same time, a shift to an autonomy-oriented focus could be said to accord respect to a pregnant woman as a moral agent and a degree of protection to the decision to terminate, conditions relevant to human flourishing and thus reasons to confer a (limited) right, as David Feldman has noted more generally in discussion of the relationship between privacy and autonomy, and also with particular reference to abortion.^153^ Erdman has likewise noted the significance of moral agency with particular reference to the decriminalisation of abortion regulation in response to a human rights critique.^154^ While the question of the relationship between privacy and autonomy is long-standing, we have seen that the Court can be said to be increasingly recognising the importance of autonomy interests, including to some extent in the abortion context.^155^ Further, their importance is embedded in the Resolution of the Parliamentary Assembly of the Council of Europe, which explicitly stated that ‘the ultimate decision on whether or not to have an abortion should be a matter for the woman concerned, who should have the means of exercising this right in an effective way’.^156^

Very importantly, recognition of a time-limited autonomy-based right would not be incompatible with an acknowledgement of the moral seriousness of abortion, as implied for instance by the US Supreme Court in *Roe v Wade* itself, with its recognition of a state interest in ‘potential life’, reaffirmed, for example, in *Planned Parenthood of Southeastern Pennsylvania v Casey*.^157^ Indeed, the Parliamentary Assembly of the Council of Europe itself stressed at the outset of its Resolution that ‘[a]bortion must, as far as possible, be avoided. All possible means compatible with women's rights must be used to reduce the number of both unwanted pregnancies and abortions'.^158^ Thus, given the morally serious nature of the decision to terminate a pregnancy, importantly it would also be appropriate for any English law reform to be accompanied by increased attention to appropriate sexual and reproductive education, information and advice and to the availability of contraception, in ways helpfully stressed—with reference to relevant evidence on the relationship between these and a reduction in the number of unwanted pregnancies, and therefore abortions—by the Parliamentary Assembly itself.^159^

The question of the time-limit of a right would, of course, require very careful thought. If this were to be 24 weeks, after this time not only would the issue of termination more likely arise because of severe medical risks to the woman (under sections 1(1)(b) and (c)) or because of issues relating to foetal disability under section 1(1)(d), but also, it may well be widely thought that the foetus has stronger moral interests, so that an unlimited legal right to abort would be inappropriate.^160^ In fact, since the great majority of terminations are conducted in the first trimester, a shorter time-limit would still make a very significant difference (such as 18, or more particularly, 13 weeks),^161^ with termination thereafter being no less restrictive than at present. Overall, the analysis has demonstrated that Convention law regarding abortion is more complex and subtle than might be supposed, as is the question of whether any given state's abortion law, however apparently liberal, is Convention-compliant, as shown here with reference to the UK.

## FUNDING

I am also very grateful to the Wellcome Trust for Research Leave that helped to support this work (Grant No 097101/Z/11/Z).

## Footnotes

1 As, for instance, in HLA Hart's Choice theory: HLA Hart, ‘Bentham on Legal Rights’ in AWB Singer (ed.), *Oxford Essays in Jurisprudence*, Second Series (OUP, Oxford, 1973) 171–9.

2 Offences Against the Person Act 1861, ss 58 and 59; Abortion Act 1967 (as amended by the Human Fertilisation and Embryology Act 1990) s 1(1)(a)–(d).

3 With regard to the law being perceived as ‘liberal’, note for instance that ‘[t]he Department of Health sets a performance indicator on access to abortion services at under ten weeks gestation to support early access to abortion and achievement of the Sexual Health Strategy standard of a three weeks maximum waiting time’. British Pregnancy Advisory Service, *Commissioning Abortion Services: A Practical Guide* (2008), 6 <http://www.bpas.org/bpashealthcare/commissioning-bpas>, last accessed 24 May 2015. Note also that most terminations are conducted on the basis of s 1(1)(a), as noted below in text and n 8.

4 As regards political criticism of the current law, note eg the unsuccessful Termination of Pregnancy Bill 2006–7, tabled by Nadine Dorries MP, which did not go beyond a first reading. See also D Fenwick, ‘The Modern Abortion Jurisprudence under Article 8 of the European Convention on Human Rights’ (2013) 12 Med L Intl 249–76, 275–6. As regards press attention, see further below.

5 See *A, B & C v Ireland*, App No 25579/05, (2011) 53 EHRR 13, para 235. The case concerned three claims, the details of which are outwith the scope of this article. However, in brief, with regard to the first and second applicants, using a negative analysis under Article 8, the Court found no breach of Article 8 ‘having regard to the right to lawfully travel abroad for an abortion with access to appropriate information and medical care in Ireland’ (para 241). In contrast, as regards the third applicant, who was concerned about a risk to her life if her pregnancy continued, the Court found a breach of Ireland's positive obligations in relation to the ‘absence of any implementing legislative or regulatory regime providing an accessible and effective procedure by which the third applicant could have established whether she qualified for a lawful abortion in Ireland in accordance with Article 40.3.3 of the Constitution’ (para 267). As noted below, Ireland's abortion law has subsequently been partially reformed.

6 Article 8(1) reads: ‘Everyone has the right to respect for his private and family life, his home and his correspondence’. Article 8(2) reads: ‘There shall be no interference by a public authority with this right except such as is in accordance with the law and is necessary in a democratic society in the interests of national security, public safety or the economic well-being of the country, for the prevention of crime and disorder, for the protection of morals, or for the protection of the rights and freedoms of others’.

7 As is widely recognised, the question of the morality of abortion is one regarding which people disagree. On many views of the moral status of abortion, the fetus has at least some claim to moral consideration, though the strength of its claims will vary according to the view held. Thus, on a potentiality account the fetus is a person with a right to life from the moment of conception. (See eg J Finnis, ‘The Rights and Wrongs of Abortion: A Reply to Judith Thomson’, (1973) 2 Phil & Pub Aff 117–145.) On a gradualist account, the greater the development of the fetus, the more serious the reasons need be to justify termination. (See eg J Feinberg, ‘Abortion’ (1979) in his *Freedom and Fulfillment* (Princeton University Press: Princeton, 1992) 37–75.) A gradualist approach is implicitly embedded in English abortion law, given the time-limit of 24 weeks for the least stringent ground of the Act, namely s 1(1)(a). In contrast, under a personhood account of fetal moral status, the fetus has no moral claims because it is not a person, for instance because it lacks rationality and self-consciousness. (See MA Warren, ‘On the Moral and Legal Status of Abortion’, (1973) 57 The Monist 43–61.) Ronald Dworkin, who adopts a gradualist account, has argued that very few people in fact think that the morality of abortion turns on whether the fetus is a person: *Life's Dominion*: *An Argument about Abortion and Euthanasia* (Harper Collins: London, 1993) 27–8.

8 In 2013 (the last year for which figures are available), the ‘vast majority’ of abortions (180,680 in total) were carried out under s 1(1)(a) (cited in text below), known as ‘Ground C’: *Abortion Statistics, England and Wales: 2013*, *Summary Information from the Abortion Notification Forms Returned to the Chief Medical Officers of England and Wales*, para 2.8. The ‘vast majority’ were performed under 13 weeks: 79% were performed at less than 10 weeks’ gestation; and 12% were performed at 10–12 weeks’ gestation. Ibid, para 2.17. Of note, abortions after 24 weeks accounted for less than 0.1% of the total (190). Ibid, para 2.19. These percentages are very similar to those in recent years.

9 And also section 1(1)(c) in fact, below.

10 See discussion below.

11 See discussion below.

12 See esp. C Zampas and JM Gher, ‘Abortion as a Human Right – International and Regional Standards’, (2008) 8/2 Hum Rights L Rev 249–94, at 277, 278, and 279. See also D Fenwick, ‘“Abortion Jurisprudence” at Strasbourg: Deferential, Avoidant and Normatively Neutral?’, (2014) 34 Legal Stud 214–41, 227–8; but note also his reservations regarding this characterisation at 229 and in Fenwick, above n 4, 265.

13 Council of Europe, *Access to Safe and L*eg*al Abortion in Europe*, Resolution 1607 (2008). Footnote 1 reads: ‘*Assembly debate* on 16 April 2008 (15th Sitting) (see Doc. 11537 rev., report of the Committee on Equal Opportunities for Women and Men, rapporteur: Mrs Gisela Wurm; and Doc. 11576, opinion of the Social, Health and Family Affairs Committee, rapporteur: Mrs Christine McCafferty). *Text adopted by the Assembly* on 16 April 2008 (15th Sitting)’. The description of the significance of the Resolution is that of Zampas and Gher, above n 12, 251.

14 In *Paton v United Kingdom* (1980) 3 EHRR 408, the Commission held, in para 19, that the fetus could not have an absolute right to life under Article 2; it also held that if it were assumed that the fetus did have a right to life during the first trimester, this would be limited by virtue of the legal ground relied on for the abortion in question (para 38). In the earlier case of *Paton v BPAS Trustees* [1979] QB 276 a husband failed to obtain an injunction restraining an abortion being carried out on his wife.

15 *Brüggemann and Scheuten v Germany* (1981) 3 EHRR 244.

16 Eg in *A, B & C v Ireland*, para 216.

17 *Brüggemann and Scheuten v Germany*, para 59.

18 Ibid, Dissenting Opinion of Mr JES Fawcett, para 1.

19 *A, B & C v Ireland*, para 216, my emphasis.

20 *RR v Poland*, App No 27617/04, (2011) 53 EHRR 31, paras 180–1, citing *Brüggemann*, *Tysic v Poland* (App No 5410/03, (2007) 45 EHRR 42) and *A, B &* C, my emphasis. See especially the Court's conclusions in paras 208–11 and its finding of a breach of Article 8 in para 214. In this case, a woman whose foetus was suspected of having a serious genetic condition was not granted timely access to prenatal genetic testing and was therefore precluded from the possibility of access to termination under Article 4(a) 1.2 of the 1993 Family Planning Act. (In *Evans v United Kingdom* (2007) App No 6339/05 (2008) 46 EHRR 34, the ECtHR held that an embryo does not have a right to life under Article 2 (para 56). The case concerned a challenge to the consent provisions of the Human Fertilisation and Embryology Act 1990, specifically whether a man should be able to withdraw his consent to the continued storage of embryos created with his former partner when, as a result of treatment for cancer of her ovaries, she wished to use those embryos and had no other way of becoming a genetic mother. The ECtHR held that there had been no violation of Articles 8 or 14.)

21 *Tysiąc v Poland*, paras 105–7.

22 For instance, on one view, although not a proxy for autonomy, privacy might be a subset of it: T Halper, ‘Privacy and Autonomy: from Warren and Brandeis to *Roe* and *Cruzan*’, 21 J Med Philos (1996) 124–35, 133. On another view, a major flaw in work on privacy has been the notion that privacy and autonomy are synonymous concepts; rather, privacy should not be confused with, and indeed is unrelated to, autonomy: WA Parent, ‘Recent Work on the Concept of Privacy’ (1983) 20 Am Philos Q 341–55, 345.

23 *Pretty v United Kingdom*, App No 2346/02, (2002) 35 EHRR 1, in which the Court found that the illegality of assisted suicide in the UK was compatible with the ECHR under Art 8(2).

24 *Tysiąc v Poland*, para 111.

25 See eg *A, B & C* itself, paras 219–42 regarding the first and second applicants.

26 *Sunday Times v United Kingdom* (1979) 2 EHRR 245.

27 See eg J Wadham and others, *Blackstone's Guide to the Human Rights Act 1998*, (Blackstone's: London, 6th edn, 2011) paras 2.54–2.64.

28 *Soering v United Kingdom* (1989) 11 EHRR 439, para 89.

29 *Tysiąc v Poland*, para 110. Here the Court found a breach of Article 8 in relation to Polish law, on the basis of a lack of a clear procedure, in the face of disagreement, to review whether the criteria for a lawful abortion on health grounds had been established.

30 Ibid, para 111.

31 Ibid, para 112.

32 Ibid, para 113, citing *Airey v Ireland* (1981) 3 EHRR 592.

33 *A, B & C v Ireland*, para 248, citing *X and Y v the Netherlands*, (1986) 8 EHRR 235. For factors concerning the state, see further para 248.

34 ‘The Court notes that in the case before it a particular combination of different aspects of private life is concerned. While the State regulations on abortion relate to the traditional balancing of privacy and the public interest, they must – in case of a therapeutic abortion – also be assessed against the positive obligations of the State to secure the physical integrity of mothers-to-be’. *Tysiąc v Poland*, para 107.

35 Ibid, para 116: ‘Once the legislature decides to allow abortion, it must not structure its legal framework in a way which would limit real possibilities to obtain it’. See also *A, B & C v Ireland*, para 249.

36 *Airey v Ireland*.

37 Fenwick, above n 4, 261, emphasis in original.

38 Fenwick, above n 12, 227; *P & S v Poland*, App No 57375/08, Judgment of October 30 2012, para 99. The case concerned a 14-year-old rape victim, P, who had great difficulty obtaining an abortion despite being legally entitled to one.

39 Offences against the Person Act 1861, ss 58 and 59.

40 Abortion Act 1967 (as amended by the Human Fertilisation and Embryology Act 1990) s 1(1)(a)–(d).

41 However, brief reference will be made to the other grounds as appropriate.

42 BMA, *First Trimester Abortion: A Briefing Paper by the BMA's Medical Ethics Committee* (ARM: London, 2007), 2.

43 Eg, *A, B & C v Ireland*, para 216.

44 *Pretty v United Kingdom*; Fenwick, above n 4, 273; Zampas and Gher, above n 12, 265.

45 House of Commons Science and Technology Committee, *Scientific Developments Relating to the Abortion Act 1967, Twelfth Report of Session 2006–07*, Vol I, 29 October 2007.

46 Ibid, para 85, footnotes referring to evidence submissions omitted.

47 Note that, in conclusion to its Terms of Reference, the Report stated: ‘As a Committee that examines scientific and technological issues, we decided that the ethical and moral issues of abortion were not within our remit. Therefore, we decided to focus on scientific and medical evidence relating to abortion, and explicitly ruled out ethical or moral issues in the published terms of reference’. Ibid, para 3.

48 BMA, above n 42, 4, citing RC Henshaw and AA Templeton, ‘Mifepristone: Separating Fact from Fiction’ (1992) 44 Drugs 531–6.

49 On the question of respect, note Ronald Dworkin's argument, above n 7, 27–8, that on both sides of the abortion debate people are really concerned with whether and why human life is ‘sacred’, coupled with the ways in which human life may be respected or disrespected.

50 In contrast, this is the case to some extent in the USA and has been the subject of judicial deliberation in, eg, *Planned Parenthood of Southeastern Pennsylvania v Casey* (1992) 120 LEd2d 674. *Casey* gave more weight to the State's interest in potential life than *Roe v Wade* (1973 35 LEd2d 147) and therefore allowed states to create laws establishing a ‘reasonable framework’ to ensure that a woman's decision to abort is ‘thoughtful and informed’ (712 and 711, respectively) holding that ‘what is at stake is the woman's right to make the ultimate decision, not a right to be insulated from all others in so doing’ (715). For extensive discussion, see especially R. Dworkin, above n 7*.*

51 J Erdman, ‘Access to Information on Safe Abortion: A Harm Reduction and Human Rights Approach’ (2011) 34 Harvard JL Gender 413–62, 445, footnote and citations omitted.

52 S Sheldon, ‘A Missed Opportunity to Reform an Outdated Law’, (2009) 4 Clinical Ethics 3–5, 4.

53 See eg *Tysiąc v Poland*, para 111, citing *Keegan v Ireland* (1994) 18 EHRR 342, para 49.

54 RCOG, *Termination of Pregnancy for Fetal Abnormality in England, Wales and Scotland* (January 1996) para 3.4, with regard to s 1(1)(a).

55 J Erdman, above n 51, 458.

56 ‘The Assembly takes the view that abortion should not be banned within reasonable gestational limits. A ban on abortions does not result in fewer abortions but mainly leads to clandestine abortions, which are more traumatic and increase maternal mortality and/or lead to abortion “tourism” which is costly, and delays the timing of an abortion and results in social inequities. The lawfulness of abortion does not have an effect on a woman's need for an abortion, but only on her access to a safe abortion’. Council of Europe, above n 13, para 4. For relevant discussion, see also Zampas and Gher, above n 12, 287–8.

57 BMA, above n 42, 2, footnote omitted. (This cites, ‘for example’: *Confidential Enquiry into Maternal and Child Health: Why Mothers Die* 2000–2002 (RCOG Press: London, 2004); Royal College of Obstetricians and Gynaecologists, *The Care of Women Requesting Induced Abortion* (RCOG Press: London, 2004); and Royal College of Obstetricians and Gynaecologists, *Thromboprophylaxis during Pregnancy, Labour and after Vaginal Delivery*, Guideline No 37 (RCOG, 2007).)

58 Ibid, 3, citing I Kennedy and A Grubb, *Medical Law* (Butterworths: London, 3rd edn, 2000) 1420.

59 A Grubb, ‘The New Law of Abortion: Clarification or Ambiguity?’ (1991) Sep Crim LR 659–70, 661.

60 BMA, above n 42, 4.

61 BMA, *Memorandum of Evidence to the Science and Technology Committee Inquiry into the Scientific Developments Relating to the Abortion Act 1967*, *August 2007* (October 2007), para 2, my emphasis.

62 Ibid, paras 11–14.

63 As cited in the House of Commons Science and Technology Committee, above n 45, para 94, my emphasis.

64 ‘In 2012, medical evidence is clear that, purely on a physical level, abortion carries less risk of maternal mortality and morbidity than does childbirth. … Thus it could be argued that any abortion carried out under Section 1(1)(a) (the ground on which 98 per cent of abortions are carried out) would always be lawful, provided the authorising doctors were acting on the basis of a good faith reliance on this medical evidence base.’ *Britain's Abortion Law: What it Says and Why* (British Pregnancy Advisory Service, May 2013) 7, <http://www.reproductivereview.org/images/uploads/Britains_abortion_law.pdf>, last accessed 24 May 2014.

65 House of Commons Science and Technology Committee, above n 45, para 95, my emphasis.

66 Ibid, para 99. There is no reference to the fetus among the list of purposes, but this may be implied in ‘any other useful purpose’. The Committee notes the evidence submissions in favour of this argument in para 93.

67 Proportionality ‘is at the heart of the necessity test’ and ‘requires a reasonable relationship between the goal pursued and the means the state has chosen to achieve that goal … the state must not use a sledgehammer to crack a nut’. J Wadham and others, above n 27, para 2.62, footnote omitted citing *Fayed v United Kingdom* (1994) 18 EHRR 393, para 71.

68 *Tysiąc v Poland*, para 116; *A, B & C v Ireland*, para 254.

69 *Tysiąc v Poland*, para 116 regarding uncertainty, and para 117 regarding formal review; *A, B & C v Ireland*, para 253 regarding uncertainty and also formal review.

70 *A, B &* C *v Ireland*, para 253, citing the interpretation of Article 40.3.3 in *Attorney General v X and Others* [1992] 1 IR 1 (‘the *X* case’).

71 Ibid.

72 Ibid.

73 Ibid, para 254, my emphasis.

74 *Tysiąc v Poland*, para 116.

75 Ibid. The Court refers to the issue of review in para 117, and to the question of time and delay in para 118.

76 *R v Smith* [1973] 1 WLR 1510. Dr Smith appealed against his conviction. On the question of whether Dr Smith had formed a good-faith opinion, Scarman LJ stated, 1517: ‘If accepted, … [the medical evidence] indicated to the jury that it was usual for a doctor to make some inquiry into a girl's background, environment, and personal history before reaching a decision. It was open to the jury to attach importance to the speed with which Dr. Smith reached his decision to terminate, the seemingly superficial nature of such examination of her mental health as he alleged he made, and the total lack of any inquiry beyond a few questions and answers when he first saw the girl’.

77 Thus, a termination on the grounds of fetal sex might be justified where, for instance, the birth of a daughter to a woman from a particular ethnic background is going to put a woman's mental and physical health at risk in the requisite sense under s (1)(1)(a), for instance due to severe family pressure to have a son. The BMA has expressed support for this view in its BMA Ethics Department, *Law and Ethics of Abortion: BMA Views* (2007) para 1.7. This BMA view has in fact been noted by the CPS, <http://www.cps.gov.uk/news/latest_news/dpp_abortion_case_fuller_reasons/>, last accessed 24 May 2015. For press coverage, see eg H Watt, C Newell and Z Khimji, ‘Abortion Investigation: Available on Demand – an Abortion if It's a Boy You Wanted’, *Daily Telegraph*, 23 February 2012, <http://www.telegraph.co.uk/health/healthnews/9099925/Abortion-investigation-Available-on-demand-an-abortion-if-its-a-boy-you-wanted.html>, last accessed 24 May 2015. However, note that, arguably potentially misleadingly, the May 2014 DH *Guidance in Relation to Requirements of the Abortion Act 1967* (discussed below), only states under the heading ‘[a]bortion on the ground of gender’: ‘Abortion on the grounds of gender alone is illegal. Gender is not itself a lawful ground under the Abortion Act (see Annex A for the lawful grounds under Section 1(1)). However, it is lawful to abort a fetus where two RMPs are of the opinion, formed in good faith, ‘that there is a substantial risk that if the child were born it would suffer from such physical or mental abnormalities as to be seriously handicapped’, and some serious conditions are known to be gender-related.’ This last sentence refers to s 1(1)(d) of the Act.

78 See CPS, above n 77.

79 CMO (Professor Dame Sally C. Davies), Letter of 23 February 2012, entitled ‘Abortion Act (as amended): Termination of Pregnancy’, CEM/CMO/2012/16, <https://https://www.cas.dh.gov.uk/ViewandAcknowledgment/ViewAlert.aspx?AlertID=101737>, last accessed 25 May 2015.

80 See <http://www.reproductivereview.org/images/uploads/Britains_abortion_law.pdf>, last accessed 24 May 2015, 54–55, where the letter of July 2 2012 is reproduced.

81 ‘This extends throughout the abortion service, to nurses, midwives and managers; and to other doctors working in gynaecology or psychiatric services, who are not directly involved in abortion care but may be called upon to provide the ‘second signature’ on HSA1 forms.’ J Bristow, ‘Introduction’, in *Britain's Abortion Law: What it Says and Why* (BPAS), above n 64, 5.

82 See CPS, above n 77.

83 Ibid, my emphasis. With reference to the DH's July 2013 ‘Guidance note for completing the HSA1 and HSA2 abortion forms’, the CPS notes that ‘even the new guidance does not indicate that each doctor must see and examine the patient before forming a view, only that he or she must have “enough evidence of the woman's circumstances to justify that they were able to form a good faith opinion that the ground for termination exists”.’ This is available at <https://www.gov.uk/government/uploads/system/uploads/attachment_data/file/226928/2013_C_Guidance_note_for_completing_the_HSA1_and_HSA2_abortion_forms.pdf>, last accessed 24 May 2015.

84 CMO (Professor Dame Sally C. Davies), Letter of 22 November 2013, entitled ‘Abortion Act (as amended): Termination of Pregnancy’, CEM/CMO/2013/16. This is available at <https://www.cas.dh.gov.uk/ViewandAcknowledgment/ViewAlert.aspx?AlertID=102075>, last accessed 24 May 2015.

85 I am grateful to Sally Sheldon for suggesting this point to me: personal communication.

86 For instance, BPAS seeks to do this in its *Britain's Abortion Law: What it Says and Why*, above n 64, at, eg, 6.

87 See CPS, above n 77, my emphasis.

88 I am grateful to Sally Sheldon for emphasising the relevance of this point to this discussion: personal communication. This facet of English law has also been recently emphasised by Emily Jackson in ‘The Legality of Abortion for Fetal Sex’, in *Britain's Abortion Law: What it Says and Why* (BPAS), above n 64, 21.

89 *A, B & C v Ireland*, para 253, my emphasis, citing *Attorney General v X and Others* [1992] 1 IR 1 (‘the *X* case’).

90 *Tysiąc v Poland*, para 38, referring to the Law on Family Planning (Protection of the Human Foetus and Conditions Permitting Pregnancy Termination) Act 1993, s 4a.

91 Ibid, para 39, referring to an Ordinance issued by the Minister of Health on January 22 1997, s 2, my emphasis.

92 *R v Smith*, my emphasis.

93 DH, *Guidance in Relation to Requirements of the Abortion Act 1967: For all those Responsible for Commissioning, Providing and Managing Service Provision* (May, 2014). This is available at <https://www.gov.uk/government/uploads/system/uploads/attachment_data/file/313459/20140509_-_Abortion_Guidance_Document.pdf>, last accessed May 24 2015.

94 Ibid, para 5, emphasis in original.

95 Ibid, para 9. It also notes: ‘Practices have come to light recently which call into question whether doctors have acted in accordance with their legal obligations under the Abortion Act. These practices include the signing of HSA1 forms by doctors before a woman has been referred, and doctors signing forms relying solely on decisions made about the woman in question by other doctors or members of the multi-disciplinary team without any other information’. Ibid, para 10.

96 Ibid, para 6.

97 Ibid, para 11.

98 Ibid.

99 Ibid, paras 12, 13 and 14, my emphasis, footnotes citing ss 19 and 71, *Good Medical Practice*, General Medical Council (2013) omitted in the last line.

100 BMA, see further above nn 57 and 61.

101 Here the *Guidance* cites the House of Commons Science and Technology Report, above n 45, no paragraph number given. However, in that Report this was listed as just one possible reason, as cited in text following n 45 above.

102 *Government Response to the Report from the House of Commons Science and Technology Committee on the Scientific Developments Relating to the Abortion Act 1967* (2007) Cm 7278, 7, citing para 99 of the Committee's Report, above n 45.

103 *Tysiąc v Poland*, para 117.

104 Ibid.

105 *RR v Poland*, paras 201–214, esp. 208–11.

106 *A, B & C v Ireland* para 253.

107 Ibid, paras 252–3.

108 Ibid, para 253.

109 A discussion of the Act, which sets out to clarify the grounds for lawful abortion in Ireland and to provide for a system of review, at the woman's request, of medical opinions in relation to her pregnancy, is outwith my current scope. For brief reference to the facts of *A, B & C*, see above n 5.

110 *A, B & C v Ireland* para 241, and above n 5; Erdman, above n 51, 460. She notes, at 461, that ‘[a] neutral and pragmatic harm reduction approach refuses to engage with abortion as a normative matter’.

111 *RR v Poland*, para 206.

112 *P & S v Poland*, para 107.

113 House of Commons Science and Technology Committee, above n 45, para 108.

114 Ibid, para 109, citing <http://www.medfash.org.uk/publications/documents/recommended_standards_for sex health services.pdf>, now available at http://www.medfash.org.uk/uploads/files/p17abl5efr149kqsu10811h21i3tt.pdf, last accessed May 24 2015.

115 Ibid, para 102.

116 General Medical Council, *Personal Beliefs and Medical Practice* (2008) para 21.

117 Ibid, para 22.

118 I do not have the scope to discuss potential discrimination issues here, but these are helpfully noted by Erdman, above n 51, 433–4, with reference also to the ECtHR's decision in *Open Door and Dublin Well Woman v Ireland*, App. Nos 14234/88 & 14235/88, 15 Eur HR Rep 244 (1992). In this case, the Court found a breach of Article 10 in relation to ‘an injunction imposed by the Irish courts on Open Door and Dublin Well Woman to restrain them from providing certain information to pregnant women concerning abortion facilities outside the jurisdiction of Ireland by way of non-directive counselling’ (at para 9); it found that ‘the injunction may have had more adverse effects on women who were not sufficiently resourceful or had not the necessary level of education to have access to alternative sources of information’ (at para 77).

119 *Tysiąc v Poland*, para 118, particularly with reference to late abortion, given the facts of the case. The question of delay is discussed by the House of Commons Science and Technology Committee, above n 45, especially in paras 88 and 101.

120 Zampas and Gher, above n 12, 279: ‘The ECtHR's Article 8 analysis [in *Tysiąc*] holds states to their procedural obligation to make abortion practically available where it is legally available. However, the *Tysiąc* decision *may* indicate the ECtHR's unwillingness to address substantive violations of human rights, even when there is a legal basis for abortion, and propensity to rely on procedural violations to remedy the wrong’. My emphasis. See also 293.

121 *Tysiąc v Poland*, para 113, my emphasis.

122 Ibid. Separate Opinion of Judge Bonello, para 2.

123 *RR v Poland*, Partly Dissenting Opinion of Judge de Gaetano, para 4. The only potentially relevant part of Art 6(1) reads: ‘In the *determination of his civil rights* and obligations or of any criminal charge against him, everyone is entitled to a fair and public hearing within a reasonable time by an independent and impartial tribunal established by law.’ My emphasis.

124 *Tysiąc v Poland*, para 67, my emphases.

125 Ibid, para 108, my emphasis.

126 Fenwick, above n 4, 265.

127 *Tysiąc v Poland*, Dissenting Opinion of Judge Borrego Borrego, para 13, citing para 104.

128 Ibid, para 13. In supposed support he cited the following passage, from para 128, of the Court's judgment: ‘Having regard to the circumstances of the case as a whole, it cannot therefore be said that … the Polish State complied with the positive obligations to safeguard the applicant's right to respect for her private life in the context of a controversy as to whether she was entitled to a therapeutic abortion’.

129 M Brazier and S Ost, *Bioethics, Medicine and the Criminal Law, Vol. 3: Medicine and Bioethics in the Theatre of the Criminal Process* (Cambridge University Press: Cambridge, 2013) 194.

130 See eg her aptly entitled ‘“Who is the Mother to Make the Judgment”: Constructions of Woman in UK Abortion Law’ (1993) 1 Fem Leg Stud 3–22; see also S Sheldon, ‘Abortion Law and the Politics of Medical Control’, in J Bridgman and S Millns (eds), *Law and Body Politics: Regulating the Female Body* (Dartmouth: Aldershot, 1995) 105–24.

131 Council of Europe, above n 13, paras 7.1 and 7.3, respectively. Para 7.3 also refers to ‘offer[ing] the conditions for a free and enlightened choice without specifically promoting abortion’.

132 BMA, above n 61, para 2.

133 Ibid.

134 Ibid*.*

135 House of Commons Science and Technology Committee, above n 45, para 99.

136 Ibid, para 99. Empirical evidence would be needed in support of this point.

137 Ibid, para 97, footnote omitted. Here it noted that ‘[t]he Government is some way from meeting its aim of all PCTs carrying out a majority of abortions by 9 weeks and eventually 70% by 9 weeks’. Footnote omitted.

138 DH, above n 8, para 2.17.

139 See further Erdman, above n 51, 458: ‘[L]iberalisation of abortion laws, including decriminalization throughout pregnancy or to a defined gestational limit, is associated with significantly decreased abortion-related harm’. Footnote citing extensive references omitted.

140 House of Commons Science and Technology Committee, above n 45, para 99.

141 Above n 7.

142 Brazier and Ost, above n 129, 194.

143 *Roe v Wade*. For an argument that *Roe* did *not* in fact create polarising conflict regarding abortion, see L Greenhouse and R Siegal, ‘Before (and After) *Roe v Wade*: New Questions About Backlash’ (2011) 120 Yale LJ 2028–87.

144 United Nations Population Division Department of Economic and Social Affairs: *Abortion Policies: A Global Review* (2002). This is available at <http://www.un.org/esa/population/publications/abortion/profiles.htm>, last accessed May 24 2015. The following citations are from the individual country reports (no page numbers available); for further details, see the full country reports. In Denmark, ‘Law No. 350 of 13 June 1973 entitles women domiciled in Denmark to undergo an abortion during the first 12 weeks of pregnancy, after the submission of an application for abortion. In addition, the woman is to be informed of the nature and risks of the procedure and of the possibilities for assistance if the pregnancy should continue to term’. In Sweden, the relevant law is the Swedish Abortion Law of June 14 1974. ‘The law, which came into effect in 1975, permits the interruption of pregnancy on request, provided there are no medical contraindications (that is, that the procedure will not seriously endanger the woman's life or health), during the first 18 weeks of pregnancy. For pregnancies between 12 and 18 weeks of pregnancy, the pregnant woman is required to discuss the abortion with a social worker’.

145 BMA, above n 61, para 2.

146 For relevant discussion see, respectively, eg, N Rhoden, ‘Trimesters and Technology: Revamping *Roe* v *Wade*’ (1986) 95 Yale LJ 639 and B Steinbock, *Life Before Birth: the Moral and L*eg*al Status of Embryos and Fetuses* (OUP: New York, 2nd edn, 2011) 46–9. This is not to suggest that the notion of viability is not complex and contested. For relatively recent discussion of relevance at the policy level, see BMA, *Abortion Time Limits: a Briefing Paper from the BMA* (May 2005) 14–17.

147 I have discussed s 1(1)(d) in detail elsewhere. See R Scott, *Choosing Possible Lives: Law and Ethics of Prenatal and Preimplantation Genetic Diagnosis* (Hart Publishing: Oxford, 2007) Ch 2.

148 The BMA has stated: ‘BMA policy is clear that any changes in relation to first trimester abortion should not adversely impact upon the availability of later abortions.’ BMA Ethics Department, above n 77, para 2.1.4.

149 Of recent historical relevance to this point, see Sheldon, above n 52.

150 Council of Europe, above n 13, para 2: ‘These restrictions have discriminatory effects, since women who are well informed and possess adequate financial means can often obtain legal and safe abortions more easily’. See also para 3: ‘The Assembly also notes that, in member states where abortion is permitted for a number of reasons, conditions are not always such as to guarantee women effective access to this right: the lack of local health care facilities, the lack of doctors willing to carry out abortions, the *repeated medical consultations* required, the time allowed for changing one's mind and the waiting time for the abortion all have the potential to make access to safe, affordable, acceptable and appropriate abortion services more difficult, or even impossible in practice’. My emphasis.

151 The bearing that empirical evidence would have on this point was noted above.

152 I leave to one side here the ‘fetal disability’ ground under section 1(1)(d). However, I agree with S Sheldon's and S Wilkinson's argument that the disability ground can be interpreted as invoking parental interests, particularly where a fetus would as a child have an impairment compatible with a life she/he thinks is worth living. S Sheldon and S Wilkinson, ‘Termination of Pregnancy for Reason of Foetal Disability: Are There Grounds for a Special Exception in Law?’ (2001) 9 Med Law Rev 85–109. See also my discussion of these issues in Scott, above n 147.

153 In connection with privacy and its links with autonomy and dignity, Feldman has written: ‘The combination of the idea of a right to be respected as a moral agent with the idea of social spheres of decision-making within which people or groups are entitled to regard themselves as free from outside coercion are, I suggest, of the essence of the notion of privacy as a civil liberty’. ‘Secrecy, Dignity, or Autonomy? Views of Privacy as a Civil Liberty’ (1994) 47 Curr Legal Problem 41–71, 55. He refers, in this connection, to the issue of abortion in his ‘Privacy-related Rights and their Social Value’ in P Birks (ed), *Privacy and Loyalty* (OUP: Oxford, 1997) 15–50, 42. Feldman argues that the weight and significance of privacy derives from its ability to facilitate the conditions for diverse forms of human flourishing (ibid, 21). For Feldman, privacy is primarily a value, rather than a right, which spawns ‘privacy-related’ rights which protect the conditions necessary for ‘valuable personal and social activity’ (ibid, 16).

154 Erdman, above n 51, 443, where she refers to women being ‘entrusted to make decisions’ and 461, where she notes that, under international human rights law, ‘[c]riminal abortion laws are interpreted to violate women's rights where they deny normative plurality on the meaning and significance of abortion’.

155 See citations from *A, B & C v Ireland* and *RR v Poland*, above nn 19 and 20, respectively.

156 Council of Europe, above n 13, para 6. See also, with respect to reform of English abortion law, Sheldon, above n 52, 4: ‘[T]he fact that abortion decisions are serious, with potentially far-reaching implications is all the more reason for believing that it is the pregnant women themselves who must make them. The women concerned are more likely to agonise over abortion decisions and they are better placed than doctors to be able to understand the implications of the decision for their own lives and the lives of those closest to them’.

157 *Planned Parenthood of Southeastern Pennsylvania v Casey*. See further above n 50. For extensive discussion, see especially R Dworkin, above n 7.

158 Council of Europe, above n 13, para 1.

159 Ibid. See paras 1, 7.5, 7.6, and 7.7. Of note, in 2013, ‘[t]he abortion rate was highest for women aged 22 (at 30 per 1,000)’. DH, above n 8, Executive Summary, p 5. See also the Council of Europe's recommendation, above n 13, para 7.8, that member states ‘promote a more pro-family attitude in public information campaigns and provide counselling and practical support to help women where the reason for wanting an abortion is family or financial pressure’.

160 *Roe v Wade* itself established a limit of 24 weeks, 35 LEd2d at 163–4. It is beyond my scope on this occasion to address the basis for the law beyond a time-limit for a right to abort.

161 The currently existing limit in Swedish law, above n 144, and the limit suggested by the BMA, above n 61, para 2, respectively. For the law to be no less restrictive than at present, s 1(1)(a) would thus need to be retained, respectively, either from 18 or 13 weeks, up to 24 weeks.

